# MITA oligomerization upon viral infection is dependent on its N-glycosylation mediated by DDOST

**DOI:** 10.1371/journal.ppat.1010989

**Published:** 2022-11-30

**Authors:** Yi Tu, Xiu-Juan Yin, Qian Liu, Shan Zhang, Jie Wang, Ben-Zhe Ji, Jie Zhang, Ming-Shun Sun, Yang Yang, Chen-Hui Wang, Lei Yin, Yu Liu

**Affiliations:** 1 State Key Laboratory of Virology, Frontier Science Center for Immunology and Metabolism, College of Life Sciences, Wuhan University, Wuhan, China; 2 Key Laboratory of Growth Regulation and Translational Research of Zhejiang Province, School of Life Sciences, Westlake University, Hangzhou, China; Nanjing Medical University, CHINA

## Abstract

The mediator of IRF3 activation (MITA, also named STING) is critical for immune responses to abnormal cytosolic DNA and has been considered an important drug target in the clinical therapy of tumors and autoimmune diseases. In the present study, we report that MITA undergoes DDOST-mediated N-glycosylation in the endoplasmic reticulum (ER) upon DNA viral infection. Selective mutation of DDOST-dependent N-glycosylated residues abolished MITA oligomerization and thereby its immune functions. Moreover, increasing the expression of Ddost in the mouse brain effectively strengthens the local immune response to herpes simplex virus-1 (HSV-1) and prolongs the survival time of mice with HSV encephalitis (HSE). Our findings reveal the dependence of N-glycosylation on MITA activation and provide a new perspective on the pathogenesis of HSE.

## Author summary

Interferons (IFNs) play critical roles in controlling viral infection. Insufficient production of IFNs leads to chronic infection and disease progression. Here, we report that DDOST is an important regulator of the MITA/STING-mediated production of antiviral type I IFNs. DDOST knockdown impairs the MITA-mediated IFN response to HSV-1 and double-stranded DNA (dsDNA). Mechanistically, DDOST mediates HSV-1-induced N-glycosylation of MITA at certain residues, which is essential to promote MITA oligomerization and consequent immune functions. Moreover, the expression of endogenous DDOST was specifically low in the brain. Increasing the expression of Ddost in the mouse brain strengthens the local immune functions and prolongs the survival time of mice with HSE. These findings reveal a regulatory mechanism of MITA oligomerization and provide one explanation for the pathogenesis of HSE, which provides a clue for developing a new antiviral drug.

## Introduction

The mediator of IRF3 activation (MITA, also known as STING, MPYS, or ERIS) is an essential adaptor protein in innate immune signaling leading to the production of type I interferon (IFN-I) and proinflammatory cytokines in response to cytosolic double-stranded DNA (dsDNA) [1–3]. Because cytosolic dsDNA can either invade pathogenic DNA or abnormally release mitochondrial DNA, the immune responses initiated to eliminate these abnormal dsDNA have been extensively studied in multiple diseases, such as viral infection, tumors, and radiotherapeutic emergency [4].

As the main cytosolic DNA sensor, cyclic GMP-AMP (cGAMP) synthase (cGAS) initiates the synthesis of the second messenger cGAMP, which subsequently binds to endoplasmic reticulum (ER)-located MITA [5,6]. MITA then undergoes oligomerization and translocates from the ER via ER-Golgi intermediate compartments (ERGIC) to perinuclear punctate structures with the help of coat protein complex II vesicles [2,7–9]. In this process, MITA recruits the kinase TBK1, which in turn phosphorylates MITA and leads to MITA degradation [10–12]. The transcription factor IRF3 is also recruited by MITA and phosphorylated by TBK1. Subsequently, activated interferon regulatory factor 3 (IRF3) enters the nucleus and initiates the transcription of IFN-Is, including IFNα and IFNβ. Secreted IFN-I functions as an autocrine/paracrine factor to further bind with IFN receptor (IFNR) on the cell surface and activate the Janus kinase (JAK)-signal transducer and activator of transcription (STAT) pathway. Eventually, the transcription factor STATs lead to the production of multiple IFN-stimulated genes (ISGs), which perform antiviral immune responses. In parallel, MITA also recruits IKK to activate the NF-κB-driven production of proinflammatory cytokines [11].

Posttranslational modification of MITA regulates its functions in various aspects. As mentioned above, TBK1-catalyzed phosphorylation of MITA leads to its degradation and attenuated IFN production. Degradation of MITA is also mediated by K48-linked polyubiquitination, catalyzed by RNF5, TRIM30α, and TRIM29. SUMOylation and deSUMOylation of MITA, catalyzed by TRIM38 and SENP2, respectively, at different stages delicately regulate the protein level of MITA to maintain proper immune responses [13–17]. The translocation of MITA from the ER to the ERGIC is also regulated by posttranslational modification. K63-linked polyubiquitination of MITA, catalyzed by RNF115 and mitochondrial E3 ubiquitin protein ligase 1 (MUL1), potentiates its translocation for further signaling [18,19]. Palmitoylation of MITA at the Golgi apparatus, catalyzed by protein palmitoyltransferases, is critical for MITA recruitment of downstream TBK1 and IRF3 [20,21]. Recently, Golgi apparatus-synthesized O-linked sulfation of glycosaminoglycans (sGAGs) was reported to be critical for MITA oligomerization and immune signal transduction [22]. Because of its critical role in multiple diseases, how MITA is regulated has attracted much research.

In the present study, we reported DDOST as a new mediator of MITA posttranslational modification upon DNA viral infection. Selective mutation of DDOST-dependent N-glycosylated residues abolished MITA oligomerization and thereby its immune functions. Furthermore, Ddost is expressed at much lower levels in the brain than in most peripheral tissues. Overexpression of Ddost in the brain effectively improves immune responses, limits the replication of HSV-1, and prolongs the survival time of mice with HSE.

## Results

### The OST complex is indispensable for antiviral IFN production

**Fig 1:**
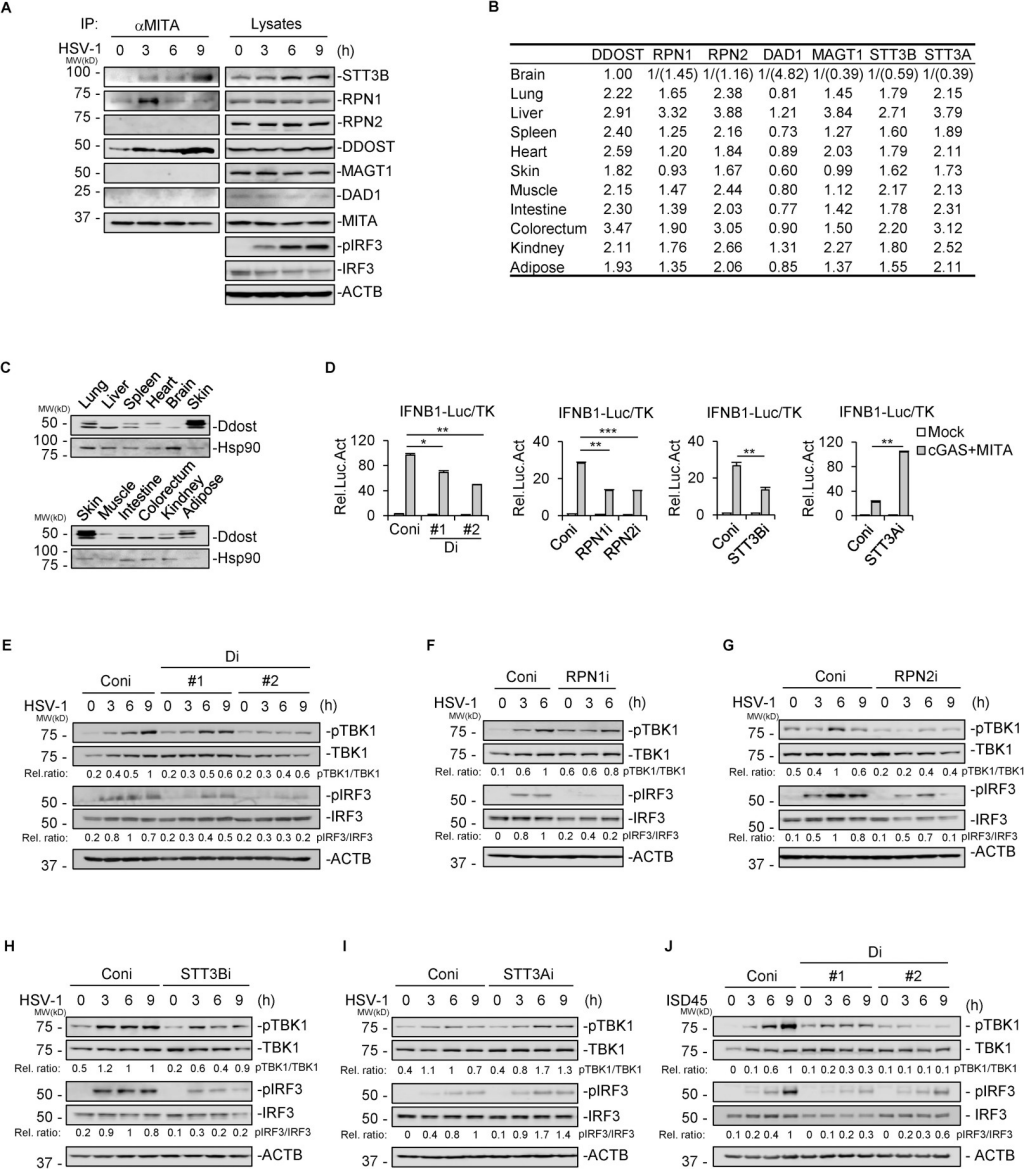
The OST complex is indispensable for antiviral IFN production. (A) THP-1 cells were infected with HSV-1 (MOI = 2) for the indicated time, and endogenous MITA was then immunoprecipitated with the indicated antibodies before immunoblotting analysis. ACTB: β-actin. (B) Consensus transcript levels of the indicated genes in human tissues were adopted from The Human Protein Atlas version 21.0 (www.proteinatlas.org) and are shown as the relative value to the transcript level of each gene in the brain. The values in the brackets represent the relative value to the transcript level of *DDOST* in the brain. (C) Different mouse organs were obtained and analyzed for the expression of Ddost by immunoblots. We discriminated proteins from humans or other species by a certain naming rule, for example, DDOST, as all letters capitalized to describe human proteins, and Ddost, as the first letter capitalized to describe mouse proteins. (D) HEK293T cells in 24-well plates were transfected with DDOST-RNAi NO.1 (Di#1), NO.2 (Di#2), RPN1-RNAi, RPN2-RNAi, STT3B-RNAi, STT3A-RNAi or empty vector control (Coni) by calcium phosphate transfection for 24 hours and then retransfected with pRK-HA-cGAS and pRK-HA-MITA together with the IFNβ firefly luciferase reporter gene (50 ng/well) and TK Renilla luciferase reporter gene (5 ng/well) by Lipofectamine 2000. A dual luciferase reporter assay was performed after 24 hours. Data displayed are the mean ± SD of each technical repeat (n = 3), *P < 0.05, **P < 0.01, ***P < 0.001 (unpaired *t* test). (E-I) Stable DDOST-, RPN1-, RPN2-, STT3A- or STT3B-knockdown THP-1 cells were constructed and infected with HSV-1 (MOI = 2) for the indicated time before immunoblotting analysis. The relative ratio (Rel. ratio) of phosphorylated TBK1 (pTBK1) to TBK1 or phosphorylated IRF3 (pIRF3) to IRF3 was calculated by measuring the grayscale values of the bands and then normalized by the value at the time of the activation peak in the control group. One representative result from at least three independent experiments is shown. (J) DDOST knockdown THP-1 cells and control cells were transfected with ISD45 (2 μg/mL) by Lipofectamine 2000 for the indicated time before immunoblotting analysis. The relative ratio (Rel. ratio) of pTBK1 to TBK1 or pIRF3 to IRF3 was calculated by measuring the grayscale values of the bands and then normalized by the value at the time of the activation peak in the control group. One representative result from at least three independent experiments is shown.

To identify potential regulators of antiviral signaling, we searched for MITA-interacting candidates in the open access shotgun mass spectrometry database (https://www.imexconsortium.org/) [23]. We found several members of the oligosaccharyltransferase (OST) complex in the database, including dolichyl-diphosphooligosaccharide-protein glycosyltransferase noncatalytic subunit (DDOST), STT3 oligosaccharyltransferase complex catalytic subunit A (STT3A), and ribophorin I (RPN1). The OST complex, which is responsible for protein glycosylation, consists of a catalytic subunit (STT3A or STT3B) and several noncatalytic subunits, including DDOST, RPN1, RPN2, defender against cell death 1 (DAD1), magnesium transporter 1 (MAGT1), oligosaccharyltransferase complex subunit 4 (OST4), transmembrane protein 258 (TMEM258), and tumor suppressor candidate 3 (TUSC3) [24–26]. We then performed endogenous coimmunoprecipitation (Co-IP) with an anti-MITA antibody and identified MITA-associated OST subunits in human monocytic THP-1 cells upon HSV-1 infection. The marked interaction between MITA and RPN1 or DDOST in the steady state was observed by immunoblotting (Fig 1A). HSV-1 infection induced the marked recruitment of STT3B to MITA and strengthened the interaction between endogenous MITA and DDOST or RPN1 (Fig 1A). However, no marked interaction between MITA and RPN2, MAGT1, or DAD1 was found. Additionally, the expression of STT3B was elevated upon HSV-1 infection, but the expression of DDOST remained at a constant level (Fig 1A). Because of the failure to detect endogenous STT3A, we overexpressed Flag-MITA and HA-STT3A plasmids in HEK293T cells and detected the interaction between MITA and STT3A by Co-IP (S1A Fig).

We then examined the expression level of OST subunits in different organs using the online database (https://www.proteinatlas.org/). The transcript abundance of *DDOST* in the brain was much lower than that in other organs, and a similar phenomenon was found in OST subunits other than *DAD1* (Fig 1B). Choosing DDOST as a representative subunit, we detected the protein levels of Ddost in different mouse organs. As shown in Fig 1C, the expression of Ddost in the brain was indeed much lower than that in the lung, liver, spleen, skin, intestine, colorectum or kidney.

Then, we sought to identify whether the low expression of the OST subunit affects MITA-mediated antiviral signaling. Specific RNA interference (RNAi) plasmids for the OST subunit were constructed, and their knockdown efficiency was confirmed by immunoblotting or qPCR (S1B–S1E Fig). Two RNAi plasmids specific to *DDOST* were named DDOST-RNAi No. 1 (Di#1) and No. 2 (Di#2). Quantified by qPCR, Di#2 inhibited the transcription level of *DDOST* to 11% of the control cells, better than Di#1 (48%) (S1B Fig). Then, IFN-β luciferase reporter assays were performed in HEK293T cells with transient transfection of RNAi plasmids. As shown in Fig 1D, knockdown of DDOST, RPN1, RPN2 or STT3B markedly inhibited cGAS-MITA-mediated activation of the IFN-β promoter, although no marked interaction between MITA and RPN2 was observed. In contrast, STT3A knockdown potentiated IFN-β promoter activation (Fig 1D), suggesting that the role of STT3A in MITA signaling differs from that of other detected subunits. Then, we prepared stable OST subunit knockdown THP-1-cell lines and infected cells with HSV-1. Consistently, DDOST, RPN1, RPN2, and STT3B knockdown inhibited HSV-1 infection-induced phosphorylation of TBK1 and IRF3 (Fig 1E–1H). In particular, both the Di#1 and Di#2 cell lines showed reduced activation of MITA signaling in response to HSV-1 compared with the control cells, and the suppression degree correlated with the knockdown efficiency of the corresponding RNAi plasmid. (Fig 1E). However, STT3A knockdown slightly promoted the phosphorylation of TBK1 and IRF3 (Fig 1I). To identify whether DDOST affects MITA signaling or virus entry directly, we transfected DDOST-knockdown THP-1 cells with double-stranded 45-mer IFN stimulatory DNA (ISD45) and observed that dsDNA-induced phosphorylation of TBK1 and IRF3 was also attenuated by DDOST knockdown (Fig 1J). In addition, DDOST knockdown impaired gradient viral infection (multiplicity of infection (MOI) = 1, 2, 4)-induced phosphorylation of TBK1 and IRF3 (S1F Fig). These data suggest that the OST complex is required for regulating MITA-mediated antiviral signaling, and the low expression of OST subunits in the brain is possibly one of the reasons for HSE. We then chose DDOST as a representative subunit to explore its antiviral functions.

### DDOST potentiates DNA-induced antiviral immune responses

**Fig 2:**
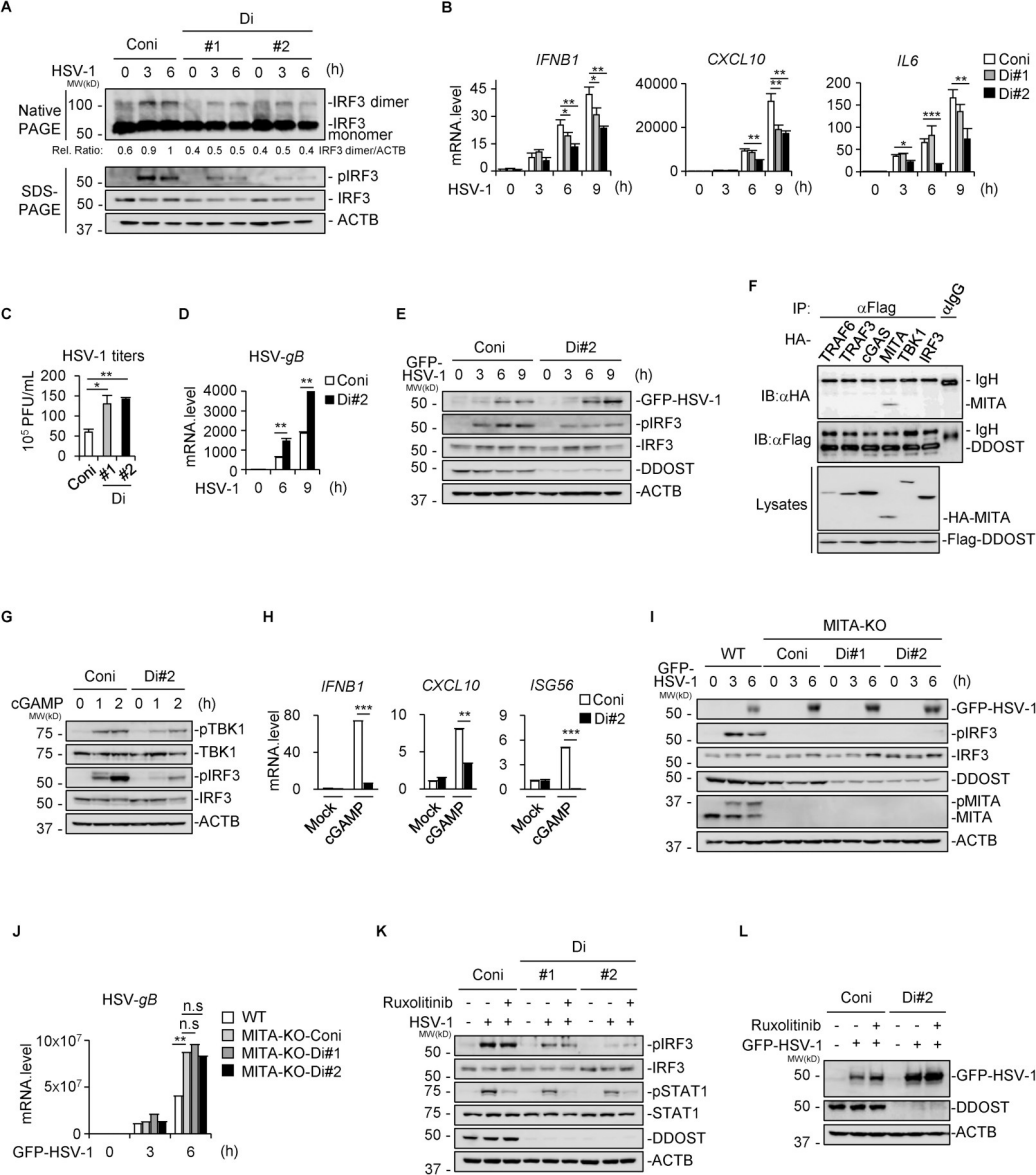
DDOST potentiates DNA-induced antiviral immune responses. (A) DDOST knockdown THP-1 cells and control cells were infected with HSV-1 (MOI = 2) for the indicated time before Native-PAGE (upper panel) and SDS–PAGE (bottom panels) analysis. The relative ratio (Rel. ratio) of the IRF3 dimer to ACTB was calculated by measuring the grayscale values of the bands and then normalized to the value at the time of the activation peak in the control group. One representative result from at least three independent experiments is shown. (B) DDOST knockdown THP-1 cells and control cells were infected with HSV-1 (MOI = 2) for the indicated time before qPCR analysis. The value from qPCR was first normalized with *ACTIN* and then divided by the normalized value of the control. Data displayed are the mean ± SD (n = 3). *P < 0.05, **P < 0.01, ***P < 0.001 (unpaired *t* test). (C) DDOST knockdown THP-1 cells and control cells were infected with HSV-1 (MOI = 1) for 24 hours before the plaque assays were performed. (D) Transcription of HSV-*gB* in transfected DDOST-knockdown and control cells was determined by qPCR. Data displayed are the mean ± SD (n = 3). **P < 0.01 (unpaired *t* test). (E) DDOST knockdown THP-1 cells and control cells were infected with GFP-HSV-1 (MOI = 2) for the indicated time before immunoblotting analysis. One representative result from at least three independent experiments is shown. (F) HEK293T cells were transfected with Flag-DDOST and HA-tagged indicated molecules before Co-IP analysis was performed. IgH: immunoglobin heavy chain. (G, H) DDOST knockdown HaCaT cells and control cells were treated with 2’,3’-cGAMP (50 ng/mL) together with digitonin (10 μg/mL) for permeabilization for the indicated time, followed by immunoblotting (G) or qPCR (H). One representative result from at least three independent experiments is shown (G). Data displayed are the mean ± SD (n = 3). **P < 0.01, ***P < 0.001 (unpaired *t* test). (I, J) Stable MITA-deficient HaCaT cells (MITA-KO) were established by the CRISPR/Cas9 technique. Endogenous DDOST in MITA-KO cells was then knocked down by lentiviral transduction (MITA-KO-Coni, MITA-KO-Di#1, MITA-KO-Di#2). These cell lines with control cells (WT) were infected with GFP-HSV-1 (MOI = 2) for the indicated time. The replication of GFP-HSV-1 was determined by immunoblotting (I) and qPCR (J). Data displayed are the mean ± SD (n = 3). **P < 0.01, (unpaired *t* test). (K, L) DDOST knockdown THP-1 cells and control cells treated with or without ruxolitinib (500 nM) were infected with HSV-1 (MOI = 2) (K) or GFP-HSV-1 (MOI = 2) (L) for the indicated time before immunoblotting analysis.

To elucidate the role of DDOST in DNA-induced antiviral immune responses, we identified whether DDOST knockdown affects the molecular events downstream of MITA upon HSV-1 infection. IRF3 dimerization upon HSV-1 infection was inhibited in DDOST knockdown THP-1 cells (Fig 2A). Consequently, the transcription of *IFNB1*, *C-X-C motif chemokine ligand 10* (*CXCL10*), and *interleukin-6* (*IL6*) in THP-1 cells upon HSV-1 infection was attenuated by DDOST knockdown (Fig 2B). We then infected THP-1 cells with HSV-1 or GFP-HSV-1 to determine whether DDOST knockdown affects HSV-1 replication. By plaque assays, we found that HSV-1 replication was promoted by DDOST knockdown (Fig 2C). The transcription of HSV-*gB* was promoted by DDOST knockdown (Fig 2D). Consistently, immunoblotting for GFP levels in GFP-HSV-1-infected cells showed that DDOST knockdown promoted viral replication (Fig 2E).

By Co-IP assays, we found that DDOST interacted with MITA but not cGAS, TBK1, IRF3, TRAF3, or TRAF6 (Fig 2F). Moreover, DDOST knockdown markedly inhibited 2′, 3′-cGAMP-induced phosphorylation of TBK1 and IRF3 and the subsequent transcription of *IFNB1*, *CXCL10*, and *IFN-stimulated gene 56* (*ISG56*) in human immortalized keratinocytes (HaCaT) cells (Fig 2G and 2H), suggesting that DDOST mainly functions at the MITA level but not the upstream cGAS level. To explore whether the role of DDOST in antiviral immune responses is dependent on MITA, we knocked out endogenous MITA in HaCaT cells (MITA-KO and control cells marked as WT) by CRISPR/Cas9 techniques (S2A and S2B Fig). Then, we knocked down the expression of DDOST in MITA-KO cells (MITA-KO-Di#1 and MITA-KO-Di#2) and infected these cell lines with control cells (WT and MITA-KO-Coni cells) with GFP-HSV-1. By immunoblotting assays, we found that GFP-HSV-1-induced phosphorylation of IRF3 was almost blocked by MITA knockout. Consistently, the expression of GFP from GFP-HSV-1 and the transcription of HSV-*gB* were significantly promoted by MITA knockout (Fig 2I and 2J). Compared with MITA-KO-Coni cells, DDOST knockdown cells (MITA-KO-Di#1 and MITA-KO-Di#2) displayed a similar pattern of antiviral immune signaling (Fig 2I). No significant difference in GFP expression and HSV-*gB* transcription was found between DDOST knockdown cells and control cells, suggesting that the function of DDOST depends on MITA (Fig 2I and 2J). To explore whether DDOST affects HSV-1 replication through the IFN-I-induced JAK-STAT signaling pathway, we treated THP-1 cells with the JAK inhibitor ruxolitinib. Ruxolitinib inhibited HSV-1-induced phosphorylation of STAT1 but not IRF3 in both DDOST knockdown cells and control cells (Fig 2K). In contrast, DDOST knockdown markedly impaired HSV-1-induced IRF3 phosphorylation but weakly attenuated STAT1 phosphorylation. The function of DDOST in IRF3 activation was hardly affected by ruxolitinib (Fig 2K). Furthermore, ruxolitinib treatment promoted the replication of GFP-HSV-1 in both DDOST knockdown cells and control cells (Fig 2L), suggesting that DDOST mainly participates in cGAS-MITA signaling. Together, these results suggest that DDOST potentiates DNA-induced antiviral responses in the cGAS-MITA signaling pathway.

### DDOST potentiates the function of MITA at the ER

**Fig 3:**
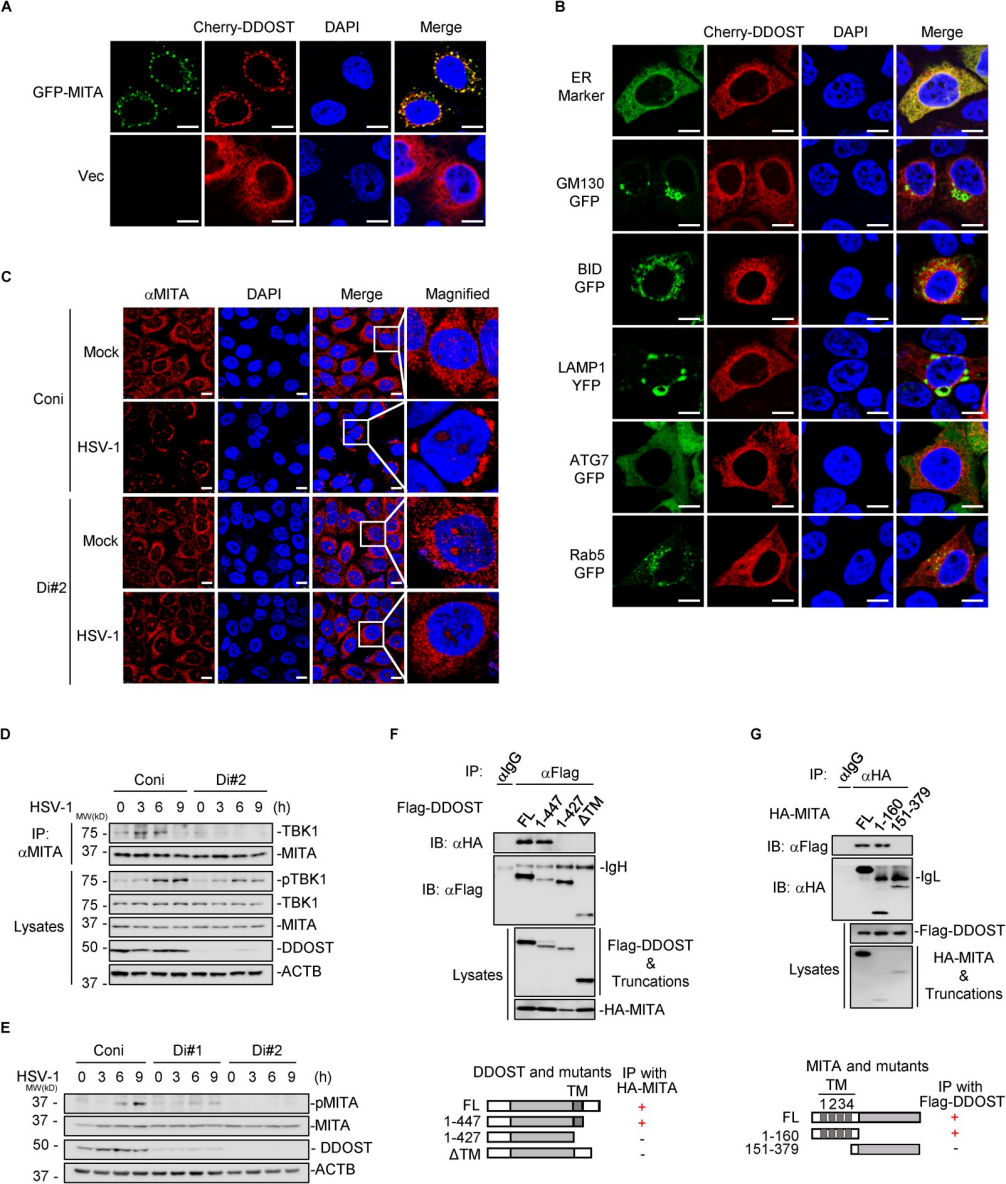
DDOST potentiates the function of MITA at the ER. (A, B) HeLa cells were transfected with Cherry-DDOST and GFP-MITA (A) or GFP-tagged organelle markers (B) and visualized under confocal microscopy 24 hours later. Vec: empty vector as a control; Scale bar: 10 μm. (C) DDOST knockdown HaCaT cells and control cells were infected with HSV-1 (MOI = 2) for 3 hours before staining with rabbit anti-MITA and Cy3 goat anti-rabbit IgG. Scale bar: 10 μm. (D) DDOST knockdown THP-1 cells and control cells were infected with HSV-1 (MOI = 2) for the indicated time. Cell lysates were subjected to Co-IP and immunoblotting analysis using the indicated antibody. (E) DDOST knockdown THP-1 cells and control cells were infected with HSV-1 (MOI = 2) for the indicated time before immunoblotting analysis. One representative result from at least three independent experiments is shown. (F, G) HEK293T cells were transfected with HA-MITA plus full length Flag-DDOST or its truncated mutants (F) or Flag-DDOST plus full length HA-MITA or its truncated mutants (G). Then, Co-IP and immunoblotting analysis were sequentially performed using the indicated antibodies 24 hours posttransfection. The related interaction diagrams are shown in the lower panels. FL: full length; TM: transmembrane domain; IgL: immunoglobin light chain.

Under fluorescence microscopy, we observed that cherry-DDOST colocalized with GFP-MITA and the ER marker CLAR but not the Golgi matrix protein GM130, mitochondrial BID, lysosomal LAMP1, or endosomal Rab5 (Fig 3A and 3B). It has been demonstrated that the diffuse distribution of MITA at the ER in the steady state aggregates to perinuclear vesicles upon DNA viral infection [2,27]. However, HSV-1-induced perinuclear aggregation of MITA was dramatically inhibited by DDOST knockdown (Fig 3C), suggesting that DDOST promotes the translocation of MITA from the ER to the perinuclear vesicles. By Co-IP assays, we confirmed that DDOST knockdown inhibited MITA recruitment of downstream TBK1 upon HSV-1 infection (Fig 3D). Consistently, HSV-1-induced phosphorylation of MITA, mainly catalyzed by TBK1, was also inhibited by DDOST knockdown (Fig 3E). These results suggest that DDOST facilitates MITA trafficking from the ER to perinuclear vesicles and thereby promotes MITA activation by recruiting TBK1.

To delineate the minimal domains responsible for DDOST-MITA interaction at the ER, we constructed a series of truncations and performed Co-IP. The results showed that DDOST and MITA interacted through their respective transmembrane domains (TM) (Fig 3F and 3G). GST pulldown assays again verified the association between DDOST and MITA (S3 Fig). Collectively, these results suggest that DDOST interacts with MITA at the ER through the TM domain and facilitates MITA trafficking from the ER to perinuclear vesicles upon viral infection.

### DDOST mediates the N-glycosylation of MITA upon viral infection

**Fig 4:**
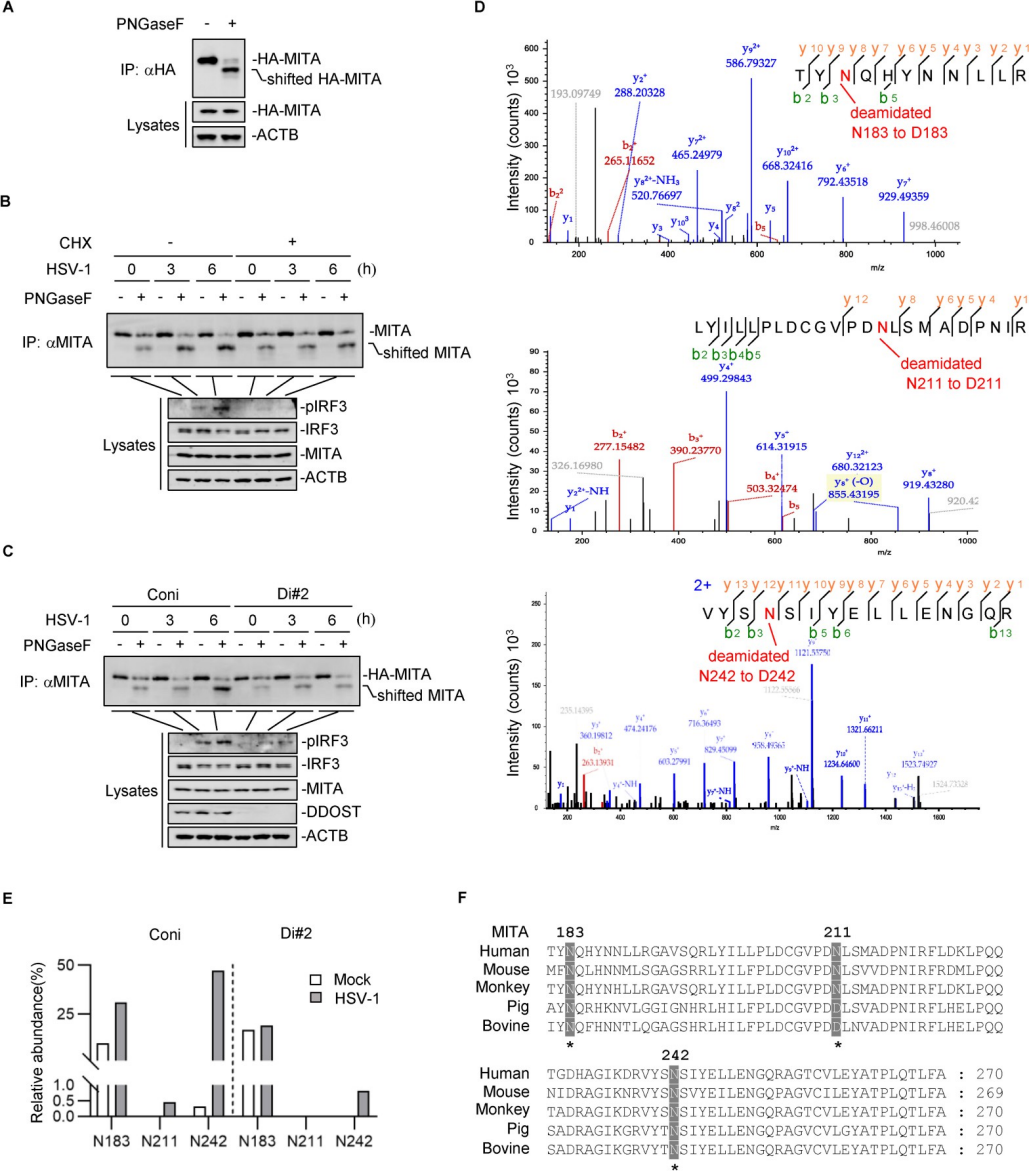
DDOST mediates the N-glycosylation of MITA upon viral infection. (A) Cell lysates of HA-MITA-transfected HEK293T cells were immunoprecipitated with an anti-HA antibody. The beads coupled with HA-MITA were incubated with PNGase F or control buffer at 37°C overnight before the immunoblotting analysis. (B) THP-1 cells were treated with or without 20 μM CHX and infected with HSV-1 (MOI = 2) for the indicated times. Then, immunoprecipitation assays were performed with an anti-MITA antibody (ABclonal, A3575) followed by digestion with PNGase F and immunoblotting. One representative result from at least three independent experiments is shown. (C) DDOST knockdown THP-1 cells and control cells were infected with HSV-1 (MOI = 2) before immunoprecipitation with anti-MITA antibody (ABclonal, A3575) and digestion with PNGase F. One representative result from at least three independent experiments is shown. (D, E) Immunoprecipitated endogenous MITA in DDOST knockdown THP-1 cells and control cells untreated or treated with HSV-1 (MOI = 2) for 6 hours was digested with PNGase F and then analyzed by LC–MS/MS to identify N-glycosylation sites of MITA. PNGase F cleaves the amide bond of N-glycosylated Asn and converts Asn to Asp. The *b*- and *y*-series fragment ions derived from a certain N-glycopeptide moiety are displayed (D). The abundance of N-glycopeptides to total peptides was calculated and displayed (E). (F) Sequence alignment of MITA homologs in humans, mice, pigs, green monkeys, and bovines.

DDOST is a noncatalytic subunit of the OST complex [28,29]. We then sought to identify whether N-glycosylation of MITA is involved in antiviral immune responses. PNGase F, as a glycosidase, catalyzes the deamidation of Asn and thereby removes the glycans attached to Asn residues in certain N-glycosylated motifs of proteins. The consequent deglycosylated proteins can be recognized by lower shifted bands in immunoblots. We immunoprecipitated ectopically expressed HA-MITA with an anti-HA antibody. The beads conjugated with HA-MITA were then incubated with PNGase F, which can catalyze the deamidation of N-glycosylated Asn. The result showed that partial MITA was deglycosylated, shifting to a lower position (Fig 4A). THP-1 cells were then infected with HSV-1 and treated with cycloheximide (CHX) to inhibit the new synthesis of proteins. Endogenous MITA was immunoprecipitated and further digested by PNGase F. The results showed that HSV-1 infection enhanced N-glycosylation of MITA, which could be mostly inhibited by CHX treatment, suggesting that virus-induced N-glycosylation of MITA mainly belongs to newly synthesized MITA (Fig 4B). Deglycosylation assays were then performed in DDOST knockdown cells. DDOST knockdown markedly reduced HSV-1-induced N-glycosylation of MITA, suggesting that DDOST is responsible for the glycosylation of newly synthesized MITA (Fig 4C).

N-glycosylation occurs on asparagine in the consensus motif Asn-X-Ser/Thr (where X is not proline) or other rare modification motifs, such as Asn-X-Cys/Val, Asn-Gly, (Ser/Thr)-X-Asn motifs and nonconsensus sequences [30,31]. We then identified the N-glycosylation sites of MITA with or without viral infection by liquid chromatography with tandem mass spectrometry (LC–MS/MS). Three residues, N183 in the Thr-X-Asn motif, N211 in the Asn-X-Ser motif, and N242 in the nonconsensus motif, were convincingly identified through three independent assays (Fig 4D). Because the total abundance of digested peptides for MS identification in DDOST knockdown cells was comparable with that in control cells, we calculated the ratio of N-glycosylated MITA peptide abundance to the total peptide abundance and found that HSV-1 infection potentiated N-glycosylation of MITA at N183, N211 and N242 (Fig 4E). However, in DDOST knockdown cells, virus-induced glycosylation at N183, N211, and N242 was inhibited, suggesting that DDOST mediates the glycosylation of MITA at these three residues (Fig 4E). Sequence alignment demonstrated that N183 and N242 of MITA were highly conserved in humans, mice, green monkeys, pigs, and bovines (Fig 4F).

### N-glycosylation at N183 and N211 is critical for MITA oligomerization

**Fig 5:**
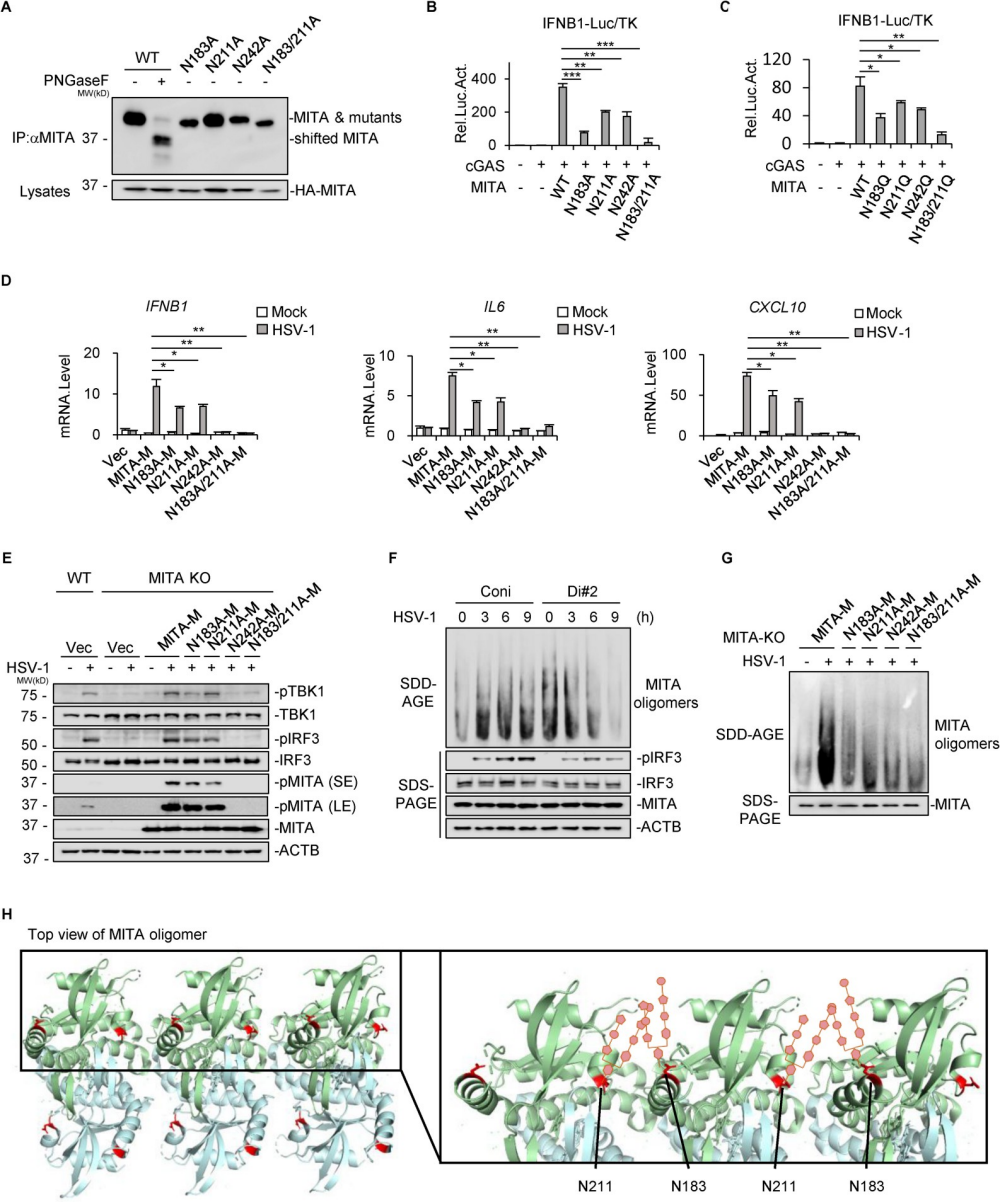
N-glycosylation of MITA at N183 and N211 is critical for its oligomerization. (A) HA-MITA (WT) or its Asn-to-Ala mutants were transfected into HEK293T cells and digested with or without PNGase F as indicated, followed by SDS–PAGE using 15% gel and immunoblotting analysis. (B, C) HA-MITA or its Asn-to-Ala mutants (B) or Asn-to-Gln mutants (C) were cotransfected with HA-cGAS and dual luciferase reporters into HEK293T cells. The activation of the IFNB1 reporter gene was measured 24 hours later. Data displayed are the mean ± SD (n = 3). *P < 0.05, **P < 0.01, ***P < 0.001 (unpaired *t* test). (D, E) Stable MITA-deficient HaCaT cells were established by transducing specific MITA gRNA. Cells were then reconstituted by transfecting CRISPR-resistant MITA mutants, including full-length MITA (MITA-M) or Asn-to-Ala mutants. Empty vector was also transfected as a control (Vec). Cells were infected with HSV-1 (MOI = 2) for 6 hours and subjected to qPCR (D) and immunoblotting analysis (E). One representative result from at least two independent experiments is shown (E). Data displayed are the mean ± SD (n = 3). *P< 0.05, **P < 0.01, ***P < 0.001 (unpaired *t* test). SE: short exposure; LE: long exposure. (F) DDOST knockdown THP-1 cells and control cells were infected with HSV-1 (MOI = 2) for the indicated time before SDD–AGE (upper panel) and SDS–PAGE (lower panel) analysis. One representative result from at least three independent experiments is shown. (G) Reconstituted HaCaT cells with MITA-M or its Asn-to-Ala mutants were infected with HSV-1 (MOI = 2) for 3 hours, followed by SDD–AGE (upper panel) or SDS–PAGE (lower panel). One representative result from at least three independent experiments is shown. (H) N-glycosylation residues are displayed in the structure of the MITA oligomer (PDB: 6DNK).

To explore the possible function of viral-induced N-glycosylation of MITA, we mutated N183, N211, and N242 to alanine or glutamic acid. The N183A and N183/211A mutants shifted to a slightly lower position than the wild-type (WT) MITA, N211A and N242A mutants (Fig 5A), suggesting that glycans attached to MITA N183 are abundant enough to affect the molecular weight and that there are some unidentified N-glycosylated sites. By dual luciferase reporter assays in HEK293T cells, we found that the activation of the IFN-β promoter by overexpressing cGAS and WT-MITA was markedly suppressed by replacing WT-MITA with Asn-to-Ala mutants, especially the N183A or N183A/N211A mutants (Fig 5B). Similar results were observed in reporter assays replacing WT-MITA with Asn-to-Gln mutants (N183Q, N211Q, N242Q and N183/N211Q) (Fig 5C). To further verify the functions of N-glycosylation at these residues in MITA signaling, we generated CRISPR-resistant MITA mutants with a one-nucleotide nonsense mutation (AGG to AAG coding for E296) in the protospacer adjacent motif following the target sequence of the gRNA plasmid. The CRISPR-resistant mutants included wild-type MITA (MITA-M) and four Asn-to-Ala mutated MITA plasmids (N183A-M, N211A-M, N242A-M, and N183/N242A-M). We then established stable reconstitution cell lines by lentiviral transduction of CRISPR-resistant mutants into MITA-KO cells and verified the antiviral effects of these mutants by HSV-1 infection. The virus-induced transcription of *IFNB1*, *IL6*, and *CXCL10* in all cells expressing Asn-to-Ala mutants was much lower than that in cells expressing MITA-M (Fig 5D). Moreover, reconstitution of N242A-M or N183A/N211A-M hardly restored the transcription of those antiviral cytokines (Fig 5D). Immunoblotting assays also confirmed that reconstitution by Asn-to-Ala mutants, especially N242A-M and N183/N211A-M, impaired HSV-1-induced phosphorylation of TBK1, MITA and IRF3 (Fig 5E). All these results demonstrate that DDOST-mediated N-glycosylation of MITA at N183, N211, and N242 is important for antiviral innate immune responses.

MITA forms obligate homodimers in the steady state in the ER and oligomerizes upon binding with cGAMP [7,8,32,33]. We then determined whether DDOST-mediated N-glycosylation of MITA contributes to MITA oligomerization at the ER. SDD–AGE was performed to detect the oligomerization of proteins as described previously [34]. HSV-1 infection induced MITA oligomerization, which was suppressed by DDOST knockdown (Fig 5F). Using reconstituted cells, we observed abundant MITA oligomerization in MITA-M-expressing cells upon HSV-1 infection, while limited MITA oligomerization was observed in N183A-M- or N211A-M-expressing cells. More importantly, MITA oligomers could hardly be seen in the N242A-M- and N183A/N211A-M-expressing cells (Fig 5G).

It has been proposed by three-dimensional reconstructions of MITA crystal structures that ligand binding induces an overlay of MITA homodimers to form linear oligomers on the same plane [7,8]. By looking into the structure of MITA oligomers presented in the PDB data, we found that N183 and N211 reside in the interface of two dimers. Because N-glycans added to the residues of proteins help establish hydrogen bonds between protein–protein interactions [35,36], the sugar chains added by N-glycosylation to N183 and N211 may fill the cavity between the dimers and help the assembly and stabilization of the oligomers, as shown in Fig 5H. It has been reported that N242 of MITA is an essential residue for MITA binding with 2′,3′-cGAMP for unknown reasons [33,37]. N242, S241, and V239 of MITA form water-mediated hydrogen bonds with O6 of the guanosine of cGAMP [37]. We therefore ascribed that DDOST-mediated N-glycosylation at N242 potentially favors the MITA-cGAMP interaction.

### Ddost overexpression strengthens antiviral immune responses of mice suffering from HSV-1 encephalitis

To explore whether mouse Ddost has a similar role as human DDOST, we designed two RNAi specific to mouse Ddost (Ddosti#1 and Ddosti#2) and transduced them into mouse lung fibroblasts (MLFs). Ddost knockdown inhibited the phosphorylation of Irf3 and Iκbα upon stimulation of HSV-1 and ISD45 (S4A and S4B Fig). However, no marked difference between Ddost knockdown cells and control cells was found in Sendai virus (SeV, known as RNA virus)-induced phosphorylation of Irf3 (S4C Fig). Consistently, HSV-1-induced transcription of antiviral cytokines *Ifnβ*, *Il6*, *Cxcl10*, and *Isg56* was markedly inhibited by Ddost knockdown (S4D Fig). These results suggest that mouse Ddost has a similar role as human DDOST in DNA virus-triggered signaling.

Mouse Ddost knockout leads to severe metabolic disorders or neurological abnormalities, and aberrant glycosylation leads to early embryonic lethality [38–40]. Because Ddost is expressed at a low level in the brain and antiviral cGAS-MITA signaling is critical for antiviral defense in the central nervous system [41], we then sought to explore whether increasing the expression of Ddost in the brain strengthens the local antiviral immune responses to DNA viruses.

**Fig 6:**
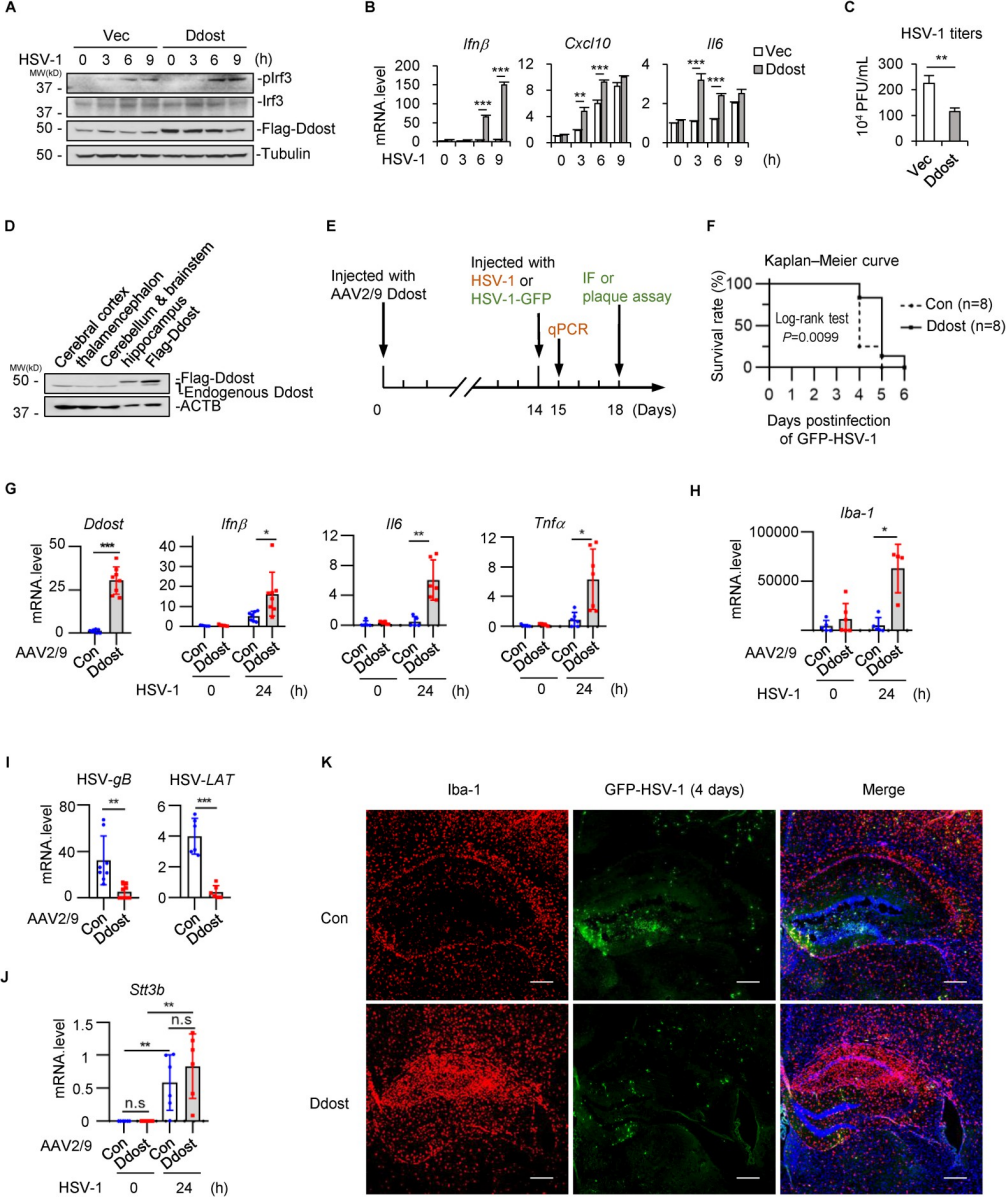
Ddost overexpression strengthens antiviral immune responses of mice suffering from HSE. (A, B) Stable mouse Neuro-2a (N2a) cells expressing Flag-Ddost and control N2a cells expressing empty vector (Vec) were prepared by lentiviral transduction and puromycin (1 μg/mL) selection for 10 days. HSV-1 (MOI = 4) was added to the cells for the indicated time, and cell lysates were collected for immunoblotting analysis (A) or qPCR (B). Data displayed are the mean ± SD (n = 3). **P < 0.01, ***P < 0.001 (unpaired *t* test). (C) Stably expressing Flag-Ddost N2a cells and control cells (Vec) were infected with HSV-1 (MOI = 2) for 24 hours. HSV-1 replication was determined by plaque assays. Data displayed are the mean ± SD (n = 3). **P < 0.01 (unpaired *t* test). (D) The expression of ectopic and endogenous Ddost in different brain regions 14 days after AAV-Ddost injection was determined by immunoblotting analysis. (E) Schematic diagram of AAV stereotactic injection and HSV-1 delivery into the mouse hippocampus region. (F) Ddost-overexpressing and control mice (n = 8 for each group) were intracranially injected with GFP-HSV-1 (2×10^5^ PFU per mouse). Mouse survival was monitored, and the survival rates were calculated and presented as Kaplan–Meier curves. (G-J) Ddost-overexpressing mice were injected with HSV-1 (3×10^4^ PFU per mouse) for 24 hours. The hippocampus was separated and analyzed by qPCR for the indicated genes. The results were normalized to *Gapdh*. Data displayed are the mean ± SD (each dot represents the result from one mouse). *P < 0.05, **P < 0.01, ***P < 0.001. (K) Ddost-overexpressing or control mice were injected with GFP-HSV-1 (3×10^4^ PFU per mouse) for 4 days (n = 5 for each group). Frozen sections (10 μm) of the hippocampus were stained with anti-Iba-1 antibody and Cy3-labeled secondary antibody. Representative confocal images are shown. Scale bar, 200 μm.

We first established mouse stable Ddost-overexpressing Neuro-2a (N2a) cells by lentiviral transduction and treated the cells with HSV-1. Overexpression of Ddost promoted the phosphorylation of Irf3 and the transcription of *Ifnβ*, *Cxcl10* and *Il6* upon HSV-1 infection and inhibited the transcription of HSV-*gB* (Figs 6A, 6B and S4E). By plaque assays, we found that Ddost overexpression inhibited the replication of HSV-1 in N2a cells 24 hours postinfection (Fig 6C). Similar results were obtained in mouse primary neuronal-glial coculture cells (S4F Fig). These data suggest that increasing the expression of Ddost potentiates antiviral MITA signaling in neurons and the consequent production of antiviral cytokines.

By immunoblotting analysis, we confirmed no marked difference in the expression of Ddost in different encephalic regions (S4G Fig). Because the most vulnerable encephalic region to HSV-1 is reported to be the hippocampus [42–44], we used an adeno-associated virus 2/9 (AAV2/9) gene transfer system to achieve Ddost overexpression in the hippocampus. The mice were randomly divided into two groups and injected with AAV2/9-Flag-Ddost and AAV2/9-empty vector plasmids. Stereotactic brain injection was performed to deliver AAV plasmids into the hippocampus. The expression of Flag-Ddost was confirmed to be specific in the hippocampus but not other encephalic regions 2 weeks after AAV injection (Fig 6D). Then, each group of mice was further divided into two groups and injected with virus or PBS as a control into the same region of the hippocampus (Fig 6E).

Lethal experiments were performed by injecting a high dose of GFP-HSV-1 into Ddost-overexpressing and control mice. By monitoring mouse survival, we found that Ddost-overexpressing mice exhibited a later onset of death (day 5 versus day 4 after infection) and a higher survival rate (*P* = 0.0099) than control mice (Fig 6F). The mortality hazard ratio of Ddost-overexpression to control calculated by the log-rank method was 0.448, which indicates that Ddost reduces the risk by 55.2% compared to the control treatment.

To explore the role of Ddost in the pathogenesis of HSV-1 encephalitis, we injected a lower dose of HSV-1 and obtained brain tissues 1 day postinjection. By qPCR analysis, we found that the transcription of antiviral cytokines *Ifnβ*, *Il6*, and *Tnfα* in the hippocampus was significantly higher in Flag-Ddost-expressing mice than in control mice (Fig 6G). Meanwhile, the viral-induced transcription of *Iba-1* and *CD68*, which are indicators of microglial cell activation, was markedly promoted by Ddost overexpression in the hippocampus (Figs 6H and S4H). Consistently, the transcription of the HSV-1 structural gene *gB* and *latency-associated transcript* (*LAT*) in the hippocampus was significantly lower in Flag-Ddost-expressing mice than in control mice (Fig 6I). To explore whether the critical catalytic subunit of the OST complex Stt3a and Stt3b participates in antiviral immune responses, we detected their expression in each group at the mRNA level. The results showed that the transcription of *Stt3b* but not *Stt3a* increased upon viral infection (Figs 6J and S4I), which is consistent with the results shown in Fig 1A that HSV-1 infection induced the expression of STT3B.

Similar viral injection was again performed using GFP-HSV-1, and samples were obtained 4 days postinjection. By frozen sections and immunofluorescence (IF) staining, we found that Iba-1-positive microglial cells were significantly recruited to the Ddost-overexpressing hippocampus but sporadically in the control group (Fig 6K). Consistently, GFP-HSV-1 particles were clustered in the control hippocampus but were much weaker and sparser in the Ddost-overexpressing hippocampus (Fig 6K). The viral titer in the brains of the Ddost-overexpressing group was markedly lower than that in the control group (S4J Fig). Collectively, these data suggest that Ddost plays an important role in host defense against DNA viruses *in vivo* and prolongs the survival time of mice with HSV-1 encephalitis by promoting the induction of type I IFNs and proinflammatory cytokines.

## Discussion

N-glycosylation is one of the critical posttranslational modification types of proteins for their stability, subcellular localization, and activities [45,46]. Aberrant glycosylation of intracellular proteins has been reported to be related to multiple diseases, such as severe inheritable metabolic syndromes, cancer, and autoimmune diseases [39,47–49]. Because MITA plays a key role in these diseases [48], it is possible that glycosylation of MITA contributes to its functions. In this study, we reported DDOST as an important regulator that potentiates MITA-mediated antiviral immune responses. DDOST knockdown impairs virus-induced N-glycosylation of MITA at N183, N211, and N242. The addition of N-glycans to these residues facilitates MITA binding with cGAMP or MITA oligomerization and thereby contributes to full MITA function.

The OST complex is the main intracellular machinery for protein N-glycosylation. In this study, we found that the OST complex containing DDOST and STT3B is responsible for viral-induced MITA N-glycosylation, which contributes to its antiviral functions based on several lines of evidence. First, DDOST and RPN1, as the main regulatory subunits of the OST complex, interact with MITA in the steady state. HSV-1 infection induces the increased expression of the catalytic subunit STT3B and induces the recruitment of STT3B to MITA. The interaction between MITA and DDOST or RPN1 is also strengthened upon HSV-1 infection. Second, viral infection increases the N-glycosylation of MITA, which is mainly a newly synthesized protein confirmed by CHX treatment. Viral-induced N-glycosylation of MITA is highly dependent on the DDOST expression level. Third, knockdown of DDOST, as well as RPN1, RPN2, and STT3B, impaired the phosphorylation of TBK1 and IRF3. Fourth, HSV-1-induced MITA trafficking and recruitment of downstream TBK1 were abolished by DDOST knockdown. The above results indicate that the DDOST and STT3B-containing OST complex is responsible for virus-induced N-glycosylation of newly synthesized MITA and contributes to full activation of MITA.

Through LC–MS/MS, we identified that DDOST knockdown impairs virus-induced N-glycosylation of MITA at N183, N211, and N242, which are conserved in several mammalian species. Mutation at these residues impaired HSV-1-induced MITA activation and subsequent production of antiviral IFNs and inflammatory cytokines. Specifically, the N242A and N183/211A mutants almost completely abolished the oligomerization and phosphorylation of MITA and the subsequent transcription of IFNs. These data demonstrated that N-glycosylation at these residues upon viral infection is critical for MITA oligomerization and its antiviral functions. Because N183 and N211 reside in the interface of two dimers of MITA, we ascribed that N-glycosylation at N183 and N211 promotes the assembly and stabilization of MITA oligomers. Previous studies have revealed that N242 of MITA is indispensable for MITA binding with cGAMP [33,37]. The glycans added to N242 may be critical for MITA binding with cGAMP.

HSV-1, as a neurotropic herpesvirus, can lead to lifelong human infection in ganglia. After primary infection and establishment of latency, HSV-1 can be reactivated upon a range of stimuli [50]. Both primary HSV-1 infection and HSV-1 reactivation can cause rare but fatal HSE, which has 70% mortality in untreated patients [51,52]. To determine the role of DDOST in individual antiviral immune responses to HSE, we first determined the antiviral functions of mouse Ddost in MLFs, N2a cells and primary neuronal-glial coculture cells and found that Ddost had a similar role to human DDOST. Because the expression of Ddost in the brain was found to be lower than that in other peripheral organs, we performed intracranial injection by AAV delivery to increase Ddost expression in the mouse hippocampus and demonstrated that overexpression of Ddost promotes the transcription of both IFNs and inflammatory cytokines in the brain. Microglial cells are markedly accumulated in the hippocampus, accompanied by diminished viral replication. Furthermore, Ddost-overexpressing mice were more resistant to a lethal dose of HSV-1. Because HSV-1 infection increases the expression of catalytic subunit STT3B of the OST complex but not DDOST, we conclude that the low expression of DDOST in the brain may be one of the reasons for insufficient local antiviral immune responses. These exciting results help elucidate the mechanism of viral encephalitis and provide valuable clues for the treatment of infectious diseases by DNA viruses.

**Fig 7:**
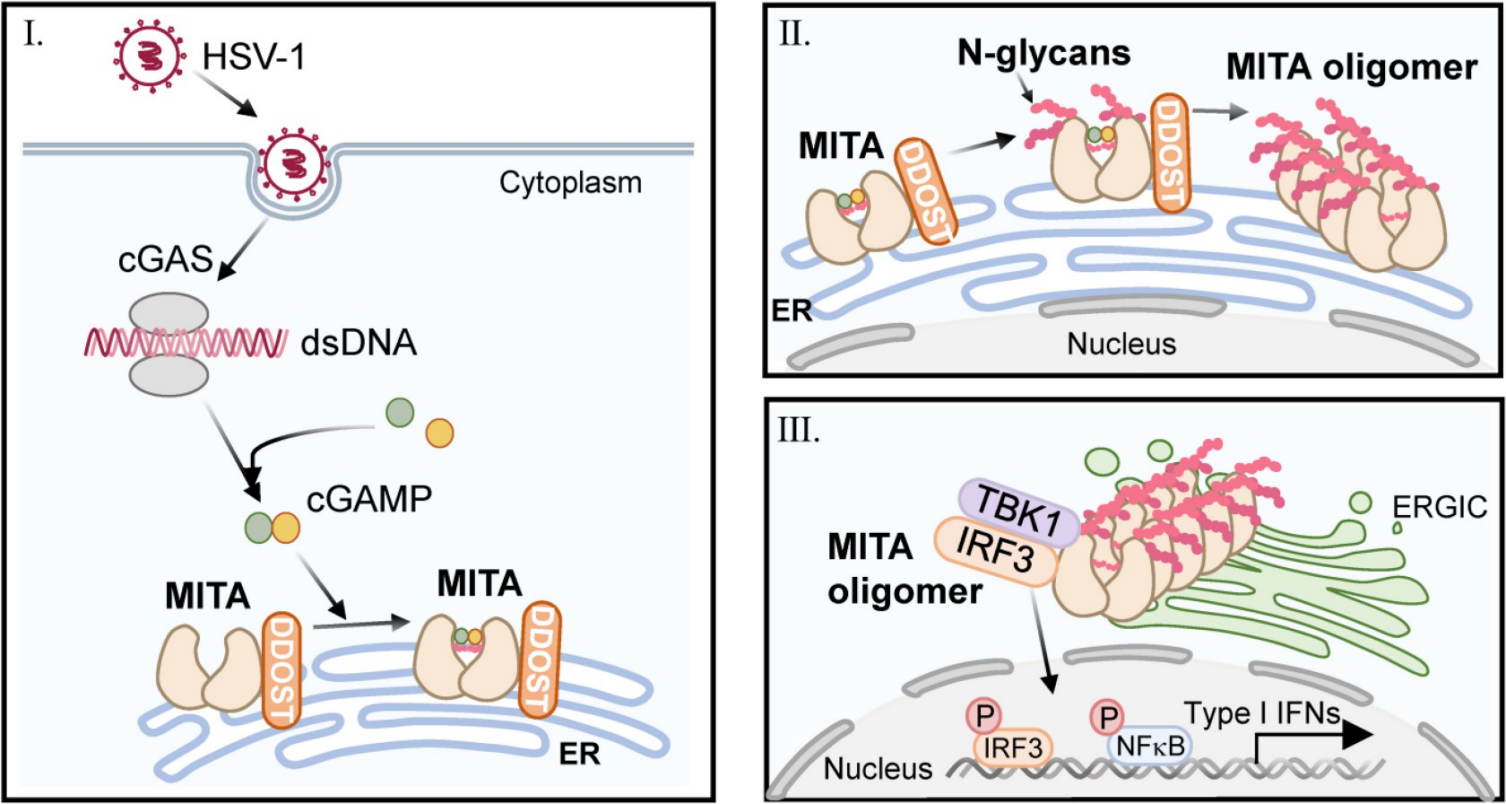
The graphical abstract of DDOST-regulated MITA-mediated signaling pathway.

In summary, we found that DNA virus-induced MITA N-glycosylation is mediated by the OST complex containing DDOST and STT3B. The identified glycosylation residues N183, N211 and N242 are necessary for MITA oligomerization and subsequent immune functions (Fig 7). Moreover, increasing the expression of Ddost in the mouse brain effectively strengthens the mouse immune response to HSV-1 and prolongs the survival time of mice with HSE. Our findings reveal the dependence of N-glycosylation on MITA activation and provide a new perspective on the pathogenesis of HSE.

## Materials and methods

### Ethics statement

All mice were maintained under SPF conditions and manipulated in the ABSL-II laboratory (G2022-001, WP20220099). All animal care and use protocols were performed in full accordance with the Regulations for the Administration of Affairs Concerning Experimental Animals approved by the State Council of People’s Republic of China and approved by the Institutional Animal Care and Use Committee of Wuhan University (SKLV AE2022-001, WP20220099).

### Antibodies and reagents

Rabbit anti-phospho-IRF3 (Ser396) (CST, 4947), anti-MITA (CST, 13647), anti-phospho-MITA (Ser366) (CST, 50907), anti-TBK1 (Abcam, ab109735), anti-phospho-TBK1 (Ser172) (Abcam, ab109272), anti-ACTB (ABclonal, AC026), anti-IRF3 (ABclonal, A19717), anti-HSP90 (Santa Cruz, sc-130068), mouse anti-phospho-IκBα (Ser32/36) (CST, 9246), anti-DDOST (Santa Cruz, sc-74408), anti-Flag (Sigma, F1804), anti-GFP (ABclonal, AE012), anti-Tubulin (ABclonal, AC012), and anti-HA (ABclonal, AE008) monoclonal antibodies were purchased from the indicated manufacturers. Rabbit anti-MITA (ABclonal, A3575), anti-RPN1 (ABclonal, A12497), anti-RPN2 (ABclonal, A8352), anti-DAD1 (ABclonal, A14723), anti-MAGT1 (ABclonal, A5039), anti-STT3B (Proteintech, 15323-1-AP), and mouse anti-IκBα (Proteintech, 10268-1-AP) polyclonal antibodies were purchased from the indicated manufacturers. Cy3 goat anti-rabbit IgG (ABclonal, AS007), goat anti-mouse IgG conjugated to HRP (Bio-Rad, 1705047), and goat anti-rabbit IgG conjugated to HRP (Bio-Rad, 1706515) were purchased from the indicated manufacturers. The dual luciferase reporter assay system (Promega, 9PIV483), Lipofectamine 2000 (Invitrogen, 11668019), 2’, 3’-cGAMP (InvivoGen, tlrl-nacga23), digitonin (Sigma–Aldrich, D141), RNAiso Plus (TAKARA, 9109), Protein G Sepharose (GE Healthcare, 17-0618-01), ABScript II cDNA First Strand Synthesis Kit (Abclonal, RK20400), 2×Universal SYBR Green Fast qPCR Mix (Abclonal, RK21203), and PNGase F (Promega, E1980) were purchased from the indicated manufacturers.

### Cells

The HSV-1-responsive human cell lines THP-1 (human leukemia monocytic cell line) and HaCaT (human keratinocyte line) were used to detect antiviral functions and perform viral replication assays. HEK293T or HeLa cells (human cervical cancer cells) were used to perform transient transfection of plasmids. Mouse lung fibroblasts (MLFs), Neuro-2a (N2a) cells, and primary neuronal-glial coculture cells were used to test the antiviral function of mouse Ddost. Primary mouse neuronal-glial cells were isolated from neonatal mouse cerebra by digestion with 0.25% trypsin and centrifugation at 200×g for 5 minutes. The precipitated neuronal-glial cells were resuspended and incubated with DMEM containing 10% FBS and 1% glutamine.

### Transfection and establishment of stable cell lines

HEK293T cells were transfected by calcium phosphate precipitation. HeLa and primary neuronal-glial coculture cells were transfected by Lipofectamine 2000. ISD45 was transfected with Lipofectamine 2000. Stable RNAi-expressing THP-1, HaCaT, MLF and N2a cells were established by infection with lentivirus packaged from HEK293T cells with psPAX2, pMD2. G, and the indicated plasmids in the pCDH-CMV-SF-IRES-Blast, pLKO.1-TRC, or lentiCRISPRv2 vectors. Stable MITA-KO HaCaT cells were generated by CRISPR/Cas9 technology. Briefly, MITA-gRNA plasmid together with packing plasmids (psPAX2 and pMD2. G) were transfected into HEK293T cells, and the packaged lentiviruses in culture medium were collected and used to infect HaCaT cells for 24 hours. The culture medium was then changed to DMEM with 10% FBS plus puromycin (1 μg/mL). Three days later, the cells were resuspended in DMEM with 10% FBS and divided into 96-well plates to obtain single clones. MITA-KO cell lines were identified by immunoblotting and genomic DNA sequencing. The stable reconstitution cell lines with CRISPR-resistant MITA mutants were generated by lentiviral transduction.

### Purification of AAV particles

HEK293T cells were transfected with pAAV-vector or pAAV-Ddost together with packaging/helper plasmids ΔF6 and AAV 2/9. The AAV particles were purified as described previously [53]. Briefly, cell lysates were suspended in gradient buffer (5 μM NaCl, 1 μM MgCl_2_ in 1 μM Tris, pH 7.6) and incubated with benzonase (50 U/mL) for 45 min at 37°C. The supernatant was applied to iodixanol gradient (15%, 25%, 40%, and 58% gradient layers) and centrifuged at 48,000 rpm for 2 hours 10 min at 16°C. Virus-containing solution enriched between the 40% and 58% gradient buffer layers was collected and washed with 0.1% F188 in PBS. The titer of purified AAV stock was determined by qPCR.

### Semidenaturing detergent agarose gel electrophoresis (SDD–AGE) assay

The SDD–AGE assay was performed as described previously [34]. Briefly, cells were completely lysed in NP-40 lysis buffer for 1 hour, and the supernatant was loaded for electrophoresis using 2×loading buffer (10% glycerol, 2% SDS, and 0.0025% bromophenol blue in 0.5×TBE buffer) at 100 V for 1 hour.

### De-N-glycosylation assay and LC–MS/MS identification

Ectopic HA-MITA or endogenous MITA in cells was first immunoprecipitated by anti-HA or anti-MITA antibody, respectively, for 4 hours at 4°C. The protein G beads coupling HA-MITA or endogenous MITA were incubated with or without PNGase F overnight at 37°C, followed by SDS–PAGE and immunoblotting. For LC–MS/MS identification, PNGase F-digested peptides were further digested by trypsin and then analyzed on an Orbitrap Exploris 480 mass spectrometer (Thermo Scientific) by SpecAlly Life Science and Technology Co., Ltd. LC–MS/MS identifies peptide residues with clear molecular weights. Because the glycosylation at Asn (MW 132.12) can be removed by PNGase F, which catalyzes the deamidation of Asn and changes it to Asp (MW 133.10), the identification of Asn-to-Asp changes in certain peptides by MS can determine the glycosylation sites of proteins.

### RNA isolation and quantitative real-time PCR (qPCR)

Total RNA from cells or tissues was extracted with RNAiso Plus and reverse transcribed to cDNA for qPCR analysis following the manufacturer’s protocol. The transcription level of target genes was normalized to the housekeeping gene *ACTIN* (for human cells) or *Gapdh* (for mouse cells or tissues).

### Co-IP and immunoblot analyses

HEK293T and THP-1 cells were lysed in NP-40 lysis buffer (150 mM NaCl, 1 mM EDTA, 1% Nonidet P-40, 10 μg/mL aprotinin, 10 μg/mL leupeptin, 1 mM phenylmethylsulfonyl fluoride in 20 mM Tris-HCl, pH 7.4). After centrifugation at 13000×g for 10 min at 4°C, the supernatant was incubated with 20 μL Protein G Sepharose and the indicated antibody or control IgG for 3 hours. The precipitate was analyzed by immunoblotting.

### Dual luciferase reporter assay

Luciferase reporter assays were performed with a dual luciferase assay kit. To normalize the transfection efficiency, 5 ng pRL-TK (*Renilla* luciferase) reporter plasmid was added to each transfection. All reporter assays were repeated at least three times.

### Virus and plaque assay

HSV-1 (ATCC VR-1789), GFP-HSV-1 (modified from HSV-1), and SeV (ATCC VR-907) were obtained from the China Center for Type Culture Collection. For plaque assays, cells or mouse brain tissues were infected with HSV-1 for 1 or 4 days. Supernatants of cultured cells or ground tissue were serially diluted and added to Vero cells for 2 hours, followed by 2% methylcellulose overlay. Two days later, Vero cells were fixed with 4% paraformaldehyde for 30 min and stained with 1% crystal violet solution before plaque counting.

### Stereotactic injection and HSV-1 intracranial infection

C57BL/6 mice were anesthetized with 0.7% (w/v) pentobarbital sodium (105 mg/kg) and fixed in a stereotactic apparatus (RWD Life Science). Referring to mouse brain stereotaxic coordinates, a tiny hole was drilled in a certain position of the skull (A/P 1.30, M/L -2.06, D/V -1.75). AAV-vector or AAV-Ddost (2.5×10^9^ viral genomes each) was injected into the left hippocampus. Two weeks later, 3×10^4^ PFU HSV-1 was reinjected at the same position. Mouse brain tissues were obtained at different days postinjection and subjected to immunoblots, qPCR, plaque assays and immunofluorescent assays. For qPCR analysis, total RNA from tissues was extracted by RNAiso Plus. Reverse transcription was then performed to synthesize cDNA per ng RNA, followed by qPCR analysis. The transcription level of target genes was normalized to the housekeeping gene *Gapdh*.

### Sequences of RNA interference (RNAi), CRISPR/Cas9 sgRNA and primers for qPCR

See S1 and S2 Tables.

### Statistical analysis and graphic preparation

The results from qPCR, luciferase reporter assays, and plaque assays were analyzed with a two-tailed unpaired *t* test by GraphPad Prism 8.0 software. The results from mouse lethal experiments were analyzed with the log-rank (Mantel–Cox) test by GraphPad Prism 8.0 software. The results from immunoblots were analyzed by ImageJ 1.40.

## Supporting information

S1 Table Sequences of RNAi and CRISPR/Cas9 gRNA. (DOCX)

S2 Table Sequences of primer sequences for RT–qPCR. (DOCX)

S1 Fig OST subunits are indispensable for dsDNA-triggered signaling pathway. (A) HEK293T were transfected with HA-STT3A and Flag-MITA followed by Co-IP analysis. (B-E) Knockdown efficiencies of the OST subunits were analyzed by qPCR or immunoblotting. (F) DDOST knockdown THP-1 cells and control cells were infected with HSV-1 (MOI = 1, 2, 4) for 6 hours before immunoblotting analysis.
(TIF)

S2 Fig N-glycosylation of MITA is critical for its activation. (A, B) MITA-deficient HaCaT cells were confirmed by immunoblotting (A) and DNA sequencing (B).
(TIF)

S3 Fig DDOST interacts with MITA directly. Purified GST-DDOST was incubated with MITA from THP-1 cell lysates, followed by Coomassie blue staining or immunoblotting.
(TIF)

S4 Fig Mouse Ddost has similar role with human DDOST in MITA-mediated innate immune responses. (A-C) Ddost knockdown MLFs and control cells were infected with HSV-1 (MOI = 1) (A), transfected with ISD45 (2 μg/ml) (B), or infected with SeV (MOI = 0.01) (C) for the indicated time before immunoblotting analysis. (D) Ddost knockdown MLFs and control cells were infected with HSV-1 (MOI = 1) for indicated times before qPCR analysis. The value from qPCR was first normalized with *Gapdh* and then divided by the normalized value of the control. Data displayed are the mean ± SD (n = 3). *P < 0.05, **P < 0.01, ***P < 0.001. (E) Stably expressing Flag-Ddost N2a cells and control cells (Vec) were infected with HSV-1 (MOI = 4) for indicated times before qPCR analysis. The value from qPCR was first normalized with *Gapdh* and then divided by the normalized value of the control. Data displayed are the mean ± SD (n = 3). ***P < 0.001. (F) Primary neuronal-glial co-culture cells were transfected with Flag-Ddost or empty vector (Vec) and infected with HSV-1 (MOI = 2) for 24 hours. HSV-1 replication was determined by plaque assays. Data displayed are the mean ± SD (n = 3). **P < 0.01. (G) The expression of AAV-Flag-Ddost from the indicated brain regions analyzed by immunoblotting. (H and I) Ddost-overexpressing mice were reinjected with HSV-1 (3×10^4^ PFU per mouse) for 24 hours. The hippocampus was separated and analyzed by qPCR for the indicated genes. Data were normalized with *Gapdh*. Data displayed are the mean ± SD (each dot represents the result from one mouse). *P < 0.05. (J) Ddost-overexpressing mice were reinjected with GFP-HSV-1 (3×10^4^ PFU per mouse) for 4 days. The hippocampus was separated and analyzed by plaque assays for GFP-HSV-1 replication. Data show the mean ± SD (each dot represents the result from one mouse). *P < 0.05.
(TIF)
