# Supplementary material for: MITA oligomerization upon viral infection is dependent on its N-glycosylation mediated by DDOST
Source: PLoS Pathog. 2022 Nov 30;18(11):e1010989. doi: 10.1371/journal.ppat.1010989 (PMC9710844; doi:10.1371/journal.ppat.1010989)
Supplement: S1 Table — (DOCX) [file ppat.1010989.s001.docx]

**S1 Table: Sequences of RNAi and CRISPR/Cas9 gRNA**

| **RNAi** | **Sequence** |
| --- | --- |
| DDOSTi#1 | 5’-GGTATTCCCAGACAGGCAACT-3’ |
| DDOSTi#2 | 5’-GCCTGTCTCTCATAAAGTATG-3’ |
| RPN1i | 5’-GCCTTTCTCACGCTATGATTA-3’ |
| RPN2i | 5’-CTGACTCAGGCCACTGTTAAA-3’ |
| STT3Ai | 5’-GCTGTAATGGTGCGTCTAATG-3’ |
| STT3Bi | 5’-GCAGGTGCTGTGTTCCTTAGT-3’ |
| Ddosti#1 | 5’-GCCTGACGTGTATGGTGTATT-3’ |
| Ddosti#2 | 5’-GCTTTGAGCTCACCTTCAAGA-3’ |
| **sgRNA** | **Sequence** |
| MITA | 5’-ACTCTTCTGCCGGACACTTG-3’ |
