# Supplementary material for: MITA oligomerization upon viral infection is dependent on its N-glycosylation mediated by DDOST
Source: PLoS Pathog. 2022 Nov 30;18(11):e1010989. doi: 10.1371/journal.ppat.1010989 (PMC9710844; doi:10.1371/journal.ppat.1010989)
Supplement: S2 Table — (DOCX) [file ppat.1010989.s002.docx]

**S2 Table: Sequences of primer sequences for RT-qPCR**

| **Target** | **Sequence** |
| --- | --- |
| *ACTIN* | Forward-5’-ACTCTTCCAGCCTTCCTTCC-3’ |
|  | Reverse-5’-CAATGCCAGGGTACATGGTG-3 |
| *IFNB1* | Forward-5’-TTGTTGAGAACCTCCTGGCT-3’ |
|  | Reverse-5’- GACTATGGTCCAGGCACAG-3’ |
| *CXCL10* | Forward-5’-GGTGAGAAGAGATGTCTGAATCC-3’ |
|  | Reverse-5’-GTCCATCCTTGGAAGCACTGCA-3’ |
| *IL6* | Forward-5’-TTCTCCACAAGCGCCTTCGGTC-3’ |
|  | Reverse-5’-TCTGTGTGGGGCGGCTACATCT-3’ |
| *ISG56* | Forward-5’- TCATCAGGTCAAGGATAGTC-3’ |
|  | Reverse-5’-CCACACTGTATTTGGTGTCTAGG-3’ |
| *DDOST* | Forward-5’-TACGCTCATCGTGGCTGACACT-3’ |
|  | Reverse-5’-CCAGCACCAAAGGGTTATCAGG-3’ |
| *Gapdh* | Forward-5’-ACGGCCGCATCTTCTTGTGCA-3’ |
|  | Reverse-5’-ACGGCCAAATCCGTTCACACC-3’ |
| *Ifnβ* | Forward-5’-TCCTGCTGTGCTTCTCCACCACA-3’ |
|  | Reverse-5’-AAGTCCGCCCTGTAGGTGAGGTT-3’ |
| *Cxcl10* | Forward-5’-ATCATCCCTGCGAGCCTATCCT-3’ |
|  | Reverse-5’-GACCTTTTTTGGCTAAACGCTTTC-3’ |
| *Il6* | Forward-5’-TCTGCAAGAGACTTCCATCCAGTTGC-3’ |
|  | Reverse-5’-AGCCTCCGACTTGTGAAGTGGT-3’ |
| *Isg56* | Forward-5’-ACAGCAACCATGGGAGAGAATGCTG-3’ |
|  | Reverse-5’-ACGTAGGCCAGGAGGTTGTGCAT-3’ |
| *Tnfα* | Forward-5’-GGTGATCGGTCCCCAAAGGGATGA-3’ |
|  | Reverse-5’-TGGTTTGCTACGACGTGGGCT-3’ |
| *Ddost* | Forward-5’-GCTGGACAACCTGAACGTG-3’ |
|  | Reverse-5’-TCCACCGACGGGGAAAAGA-3’ |
| *Iba-1* | Forward-5’-ATCAACAAGCAATTCCTCGATGA-3’ |
|  | Reverse-5’-CAGCATTCGCTTCAAGGACATA-3’ |
| *CD68* | Forward-5’-ATCCCCACCTGTCTCTCTCA-3’ |
|  | Reverse-5’-TTGCATTTCCACAGCAGAAG-3’ |
| HSV*-gB* | Forward-5’-CGCATCAAGACCACCTCCTC-3’ |
|  | Reverse-5’-AGCTTGCGGGCCTCGTT-3’ |
| HSV*-LAT* | Forward-5’-ACCCACGTACTCCAAGAAGGC-3’ |
|  | Reverse-5’-TAAGACCCAAGCATAGAGAGCCA-3’ |
