# Supplementary figures and images for: MITA oligomerization upon viral infection is dependent on its N-glycosylation mediated by DDOST
Source: PLoS Pathog. 2022 Nov 30;18(11):e1010989. doi: 10.1371/journal.ppat.1010989 (PMC9710844; doi:10.1371/journal.ppat.1010989)

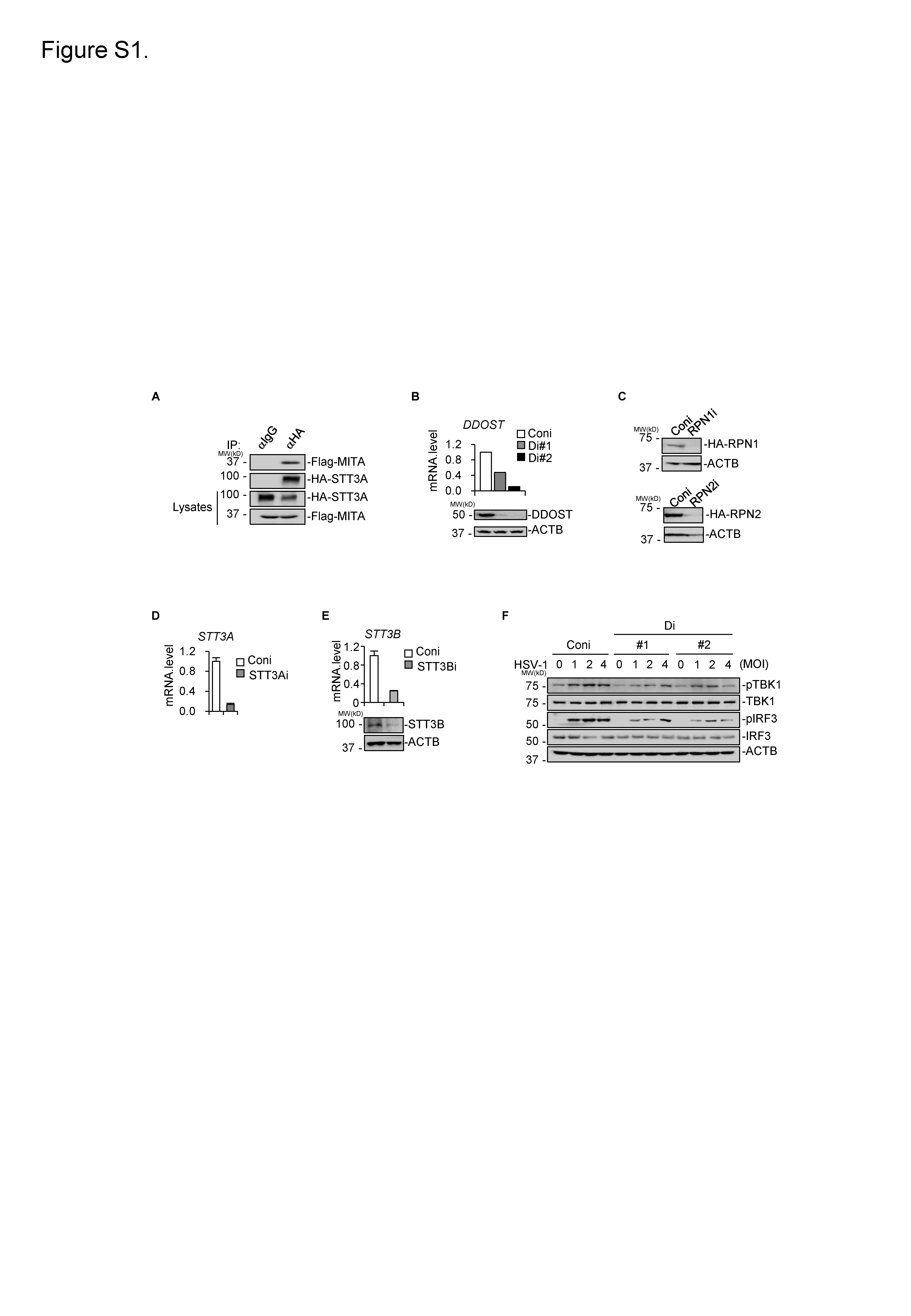

Supplement: S1 Fig — (A) HEK293T were transfected with HA-STT3A and Flag-MITA followed by Co-IP analysis. (B-E) Knockdown efficiencies of the OST subunits were analyzed by qPCR or immunoblotting. (F) DDOST knockdown THP-1 cells and control cells were infected with HSV-1 (MOI = 1, 2, 4) for 6 hours before immunoblotting analysis. (TIF) [file ppat.1010989.s003.tif]

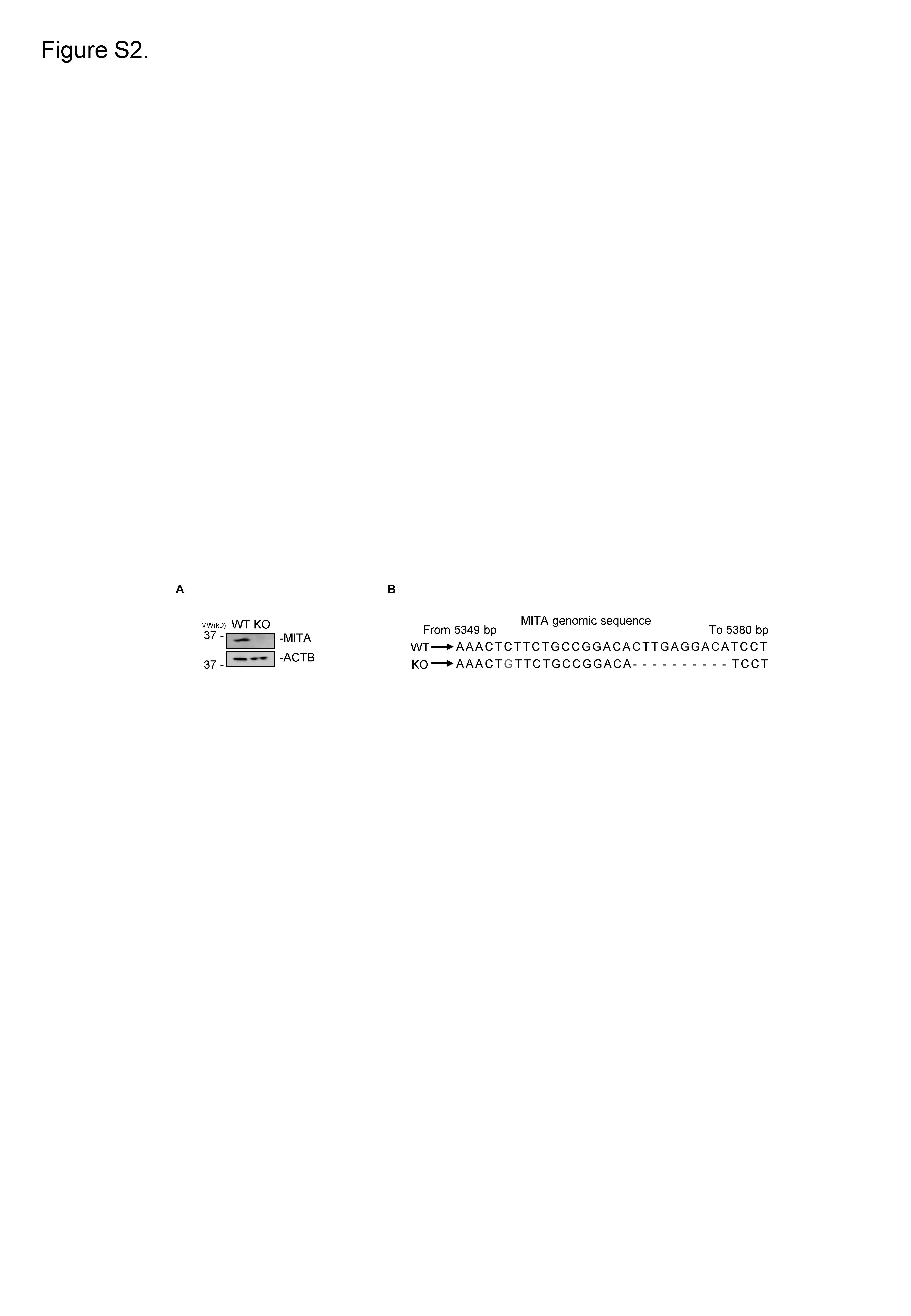

Supplement: S2 Fig — (A, B) MITA-deficient HaCaT cells were confirmed by immunoblotting (A) and DNA sequencing (B). (TIF) [file ppat.1010989.s004.tif]

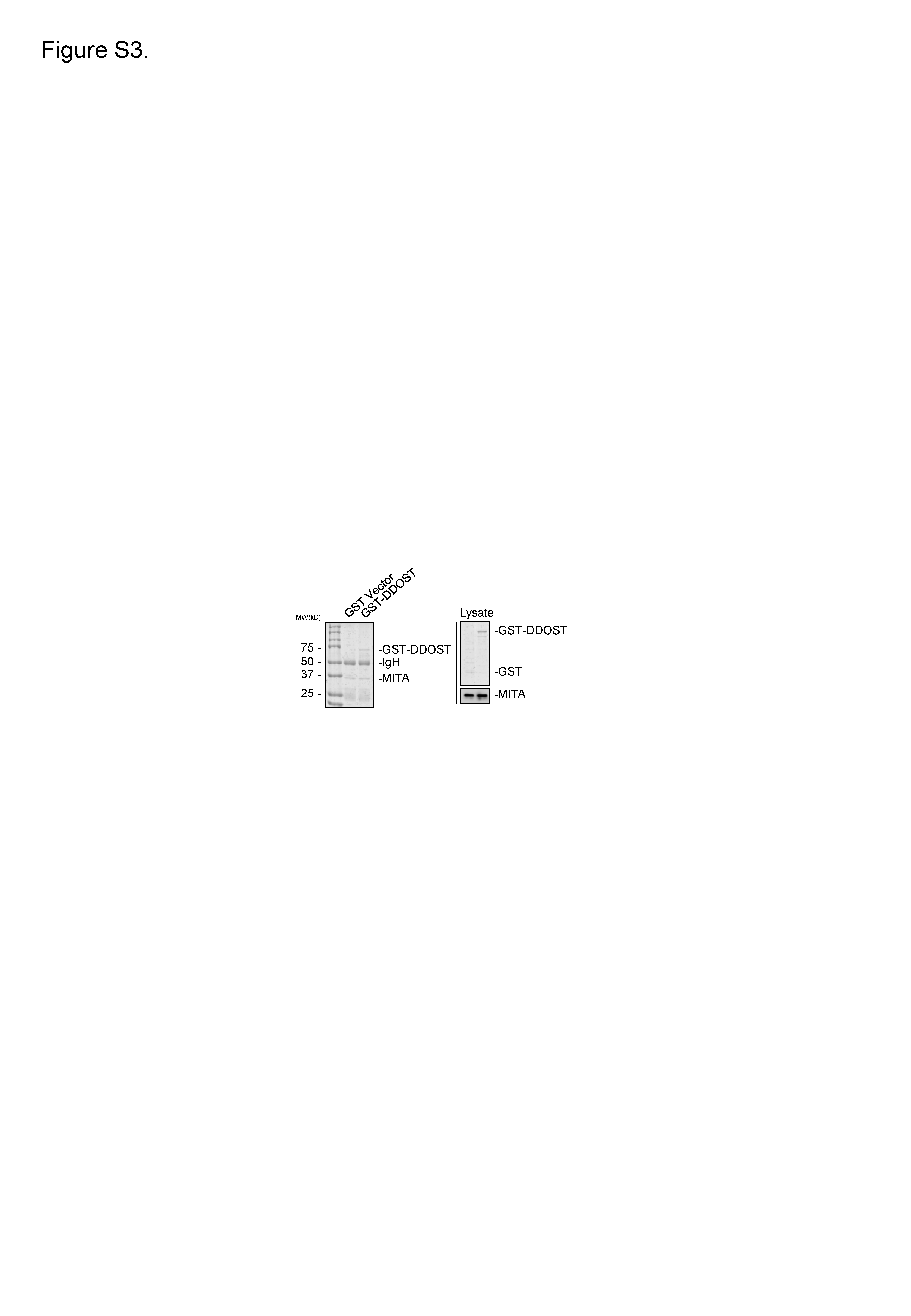

Supplement: S3 Fig — Purified GST-DDOST was incubated with MITA from THP-1 cell lysates, followed by Coomassie blue staining or immunoblotting. (TIF) [file ppat.1010989.s005.tif]

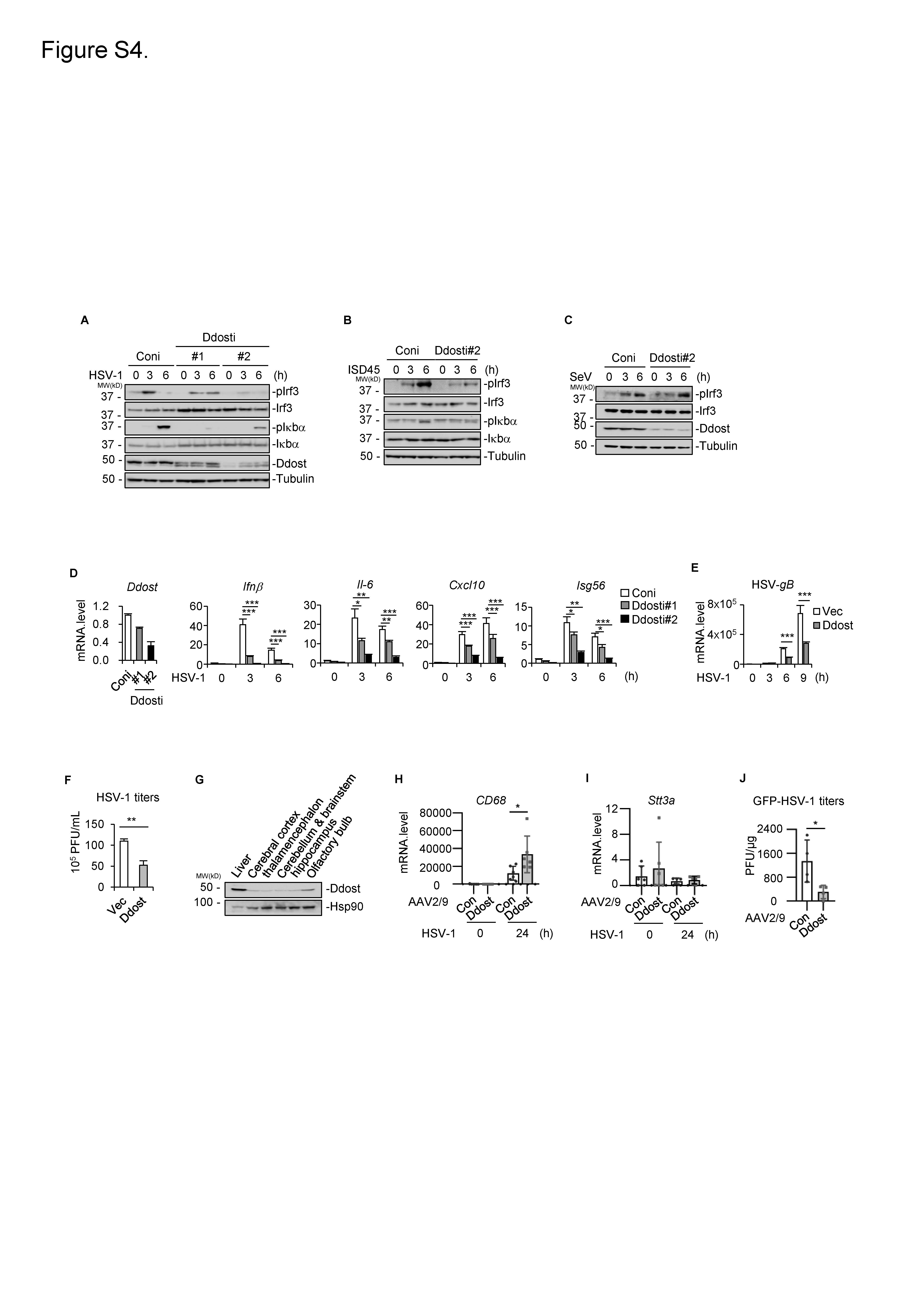

Supplement: S4 Fig — (A-C) Ddost knockdown MLFs and control cells were infected with HSV-1 (MOI = 1) (A), transfected with ISD45 (2 μg/ml) (B), or infected with SeV (MOI = 0.01) (C) for the indicated time before immunoblotting analysis. (D) Ddost knockdown MLFs and control cells were infected with HSV-1 (MOI = 1) for indicated times before qPCR analysis. The value from qPCR was first normalized with Gapdh and then divided by the normalized value of the control. Data displayed are the mean ± SD (n = 3). *P < 0.05, **P < 0.01, ***P < 0.001. (E) Stably expressing Flag-Ddost N2a cells and control cells (Vec) were infected with HSV-1 (MOI = 4) for indicated times before qPCR analysis. The value from qPCR was first normalized with Gapdh and then divided by the normalized value of the control. Data displayed are the mean ± SD (n = 3). ***P < 0.001. (F) Primary neuronal-glial co-culture cells were transfected with Flag-Ddost or empty vector (Vec) and infected with HSV-1 (MOI = 2) for 24 hours. HSV-1 replication was determined by plaque assays. Data displayed are the mean ± SD (n = 3). **P < 0.01. (G) The expression of AAV-Flag-Ddost from the indicated brain regions analyzed by immunoblotting. (H and I) Ddost-overexpressing mice were reinjected with HSV-1 (3×10^4^ PFU per mouse) for 24 hours. The hippocampus was separated and analyzed by qPCR for the indicated genes. Data were normalized with Gapdh. Data displayed are the mean ± SD (each dot represents the result from one mouse). *P < 0.05. (J) Ddost-overexpressing mice were reinjected with GFP-HSV-1 (3×10^4^ PFU per mouse) for 4 days. The hippocampus was separated and analyzed by plaque assays for GFP-HSV-1 replication. Data show the mean ± SD (each dot represents the result from one mouse). *P < 0.05. (TIF) [file ppat.1010989.s006.tif]
